# Establishment of galenic laboratories in developing countries to produce high quality medicines: results of Aid Progress Pharmacist Agreement (*A.P.P.A.*^®^) Project

**DOI:** 10.3325/cmj.2014.55.662

**Published:** 2014-12

**Authors:** Francesca Baratta, Antonio Germano, Gaetano Di Lascio, Richard Petieau, Paola Brusa

**Affiliations:** 1 Department of Scienza e Tecnologia del Farmaco, University of Turin, Turin, Italy; 2 Aid Progress Pharmacist Agreement, Turin, Italy; 3 Centre Médico-Chirurgical Maternité “la Bethanie”, Douala, Cameroon

## Aid Progress Pharmacist Agreement Project: aims in developing countries

Aid Progress Pharmacist Agreement *(A.P.P.A.^®^*) is a non-profit association based on voluntary work (1) and its main activity is the *A.P.P.A.^®^* Project. The Project started in 2005 as a result of the cooperation between the Pharmacy Faculty of Turin and Italian community pharmacists. Its main task is the establishment of galenic laboratories (GLs) in hospitals of developing countries (DCs) according to the principles of international health cooperation.

Aims of the Project:

• establishing GLs in DCs with the aim of preparing medicinal products that comply with quality requirements, first of all to fight the widespread counterfeiting of medicines in DCs;

• tailoring the dosages and pharmaceutical forms according to the actual patient needs;

• employing local staff, teaching them a “new job,” and opening a suitable school;

• minimizing the costs necessary to prepare these medicines.

There are important reasons why galenics should be used:

i) low cost of the production system and the simple operative procedures;

ii) the possibility to adapt dosages and pharmaceutical forms to the patients’ needs and medical prescriptions;

iii) reduction in the use of counterfeit medicines in the settings where the GL is located.

The Project complies both with EU and participating countries' legislation, while safeguarding the standard of medicinal products. It is the only project related to galenics research and development in the field of international health cooperation and the only one that involves close cooperation between the community pharmacies and the University. Many pharmacists have spent a lot of time doing humanitarian work in DCs but their efforts have not been long lasting. The collaboration with the University ensures the continuity of the Project and the constant quality control of the medicinal products. This guarantees that people living in DCs will use quality, safe, and efficacious medicines, rather than potentially counterfeit industrial medicinal products.

The problem of counterfeit medicines is especially serious in DCs, where custom procedures are less stringent, competent authorities’ controls are less effective, and the use of ineffective medicines may result in a substantial loss of public confidence in the health care system (2-10). The World Health Organization (WHO) defines counterfeit drugs as medicines that are deliberately and fraudulently mislabeled with respect to identity and/or source. Counterfeit medicines include products with the correct ingredients, but also with wrong ingredients, without active ingredients, with insufficient or excessive amount of active ingredient, or with false or misleading labeling (11).

A part of the *A.P.P.A.*^®^ Project is to investigate the quality of medicines purchased on site from pharmacies or unofficial street-pharmacists. This kind of investigation also helps to identify whether a setting is a potential candidate for *A.P.P.A.*^®^ Project: if drugs found on a particular market are of sufficient quality, there is no need for GL establishment.

A previous survey (12) collected samples in different DCs and analyzed them in the University of Turin laboratories. Most of the samples were manufactured in India. In fact, DCs usually import medicines as they have no production facilities and do not check their quality before distribution (13). We estimated that 50% of the tested items were substandard drugs and 2% were counterfeits without declared active pharmaceutical ingredients (APIs) (12).

## The operating steps of the *A.P.P.A.^®^* Project

The location for establishment of a new *A.P.P.A.^®^* laboratory is selected on the basis of a specific request received from a DC health care facility. The usefulness of establishing a GL in a particular setting is assessed in a preliminary study. The study implies a trip of an *A.P.P.A.^®^* staff member to the site to assess the local situation and recipient areas (step 0 of A.P.P.A.® Project). A precise protocol is used to obtain the preliminary information at this stage. Some medicines are purchased on site and sent to the laboratories of the University of Turin, where qualitative and quantitative analysis is performed to assess their compliance with the stated characteristics. The feasibility study for a new *A.P.P.A.^®^* Project consists of several stages and is specifically aimed at:

• finding a location for the galenic laboratory;

• identifying a partner for economic cooperation;

• acquainting the research group with the climatic conditions (temperature and humidity) of the area of interest;

• establishing contacts with the local medical doctors (MDs) and the head of the host health facility;

• analyzing the most frequent endemic diseases in collaboration with the MDs in charge;

• gathering information on the catchment area of the health facility;

• identifying local actors, preferably already part of the health sector, to be taught by *A.P.P.A.^®^* students during their internships on site;

• ensuring a facility (minimum 30 m^2^) equipped with running water and electricity (with the possibility of installing an air conditioner);

• assessing the possibility of buying substances (active ingredients and excipients) and equipment, or the possibility of importing them by analyzing customs restrictions and identifying the person responsible for the operation;

• analyzing local medicines to assess their quality standards and prices.

This information enables us:

• to conduct a pharmacoeconomic survey;

• to evaluate the feasibility of the Project;

• to prepare a specific handbook suited to the local needs;

• to prepare teaching materials for operators training.

After the feasibility study the *A.P.P.A.^®^* Project can start. The following six steps enable us to establish an effective and functional laboratory:

1. choosing the GL location. The Chief MD in charge of the medical center lists the local diseases and the correct pharmaceutical forms are planned;

2. at the galenic A.P.P.A.^®^ laboratory at the University of Turin, Pharmacy students are taught how to make the required medicinal products during the preparation of their experimental thesis;

3. a local staff member visits the laboratory in Turin to learn the product preparation (about one month’s work) under the supervision of Italian Pharmacy students. During this period the material for the GL is sent to the DC hospital;

4. the local staff member who visited Turin undergoes a two-month training period at the DC hospital, coordinated by Italian Pharmacy students;

5. quality control of medicinal products prepared in the new GL; some of the samples are sent to the University of Turin to be quality tested;

6. regular 40-day or longer internships for Pharmacy students from Italy organized each year to supervise the work of the laboratory and to study new formulations made according to the Chief MD’s demands.

## Established laboratories

After 8 years of work and due to the effort of about 90 people, including graduates, undergraduates, and laboratory technicians 8 operating laboratories were established, all at the step 6 (1):

• Centre Médico-Chirurgical Maternité “la Bethanie”, Douala, Cameroon, established in 2006.

• Hôpital “Notre Dame des Apòtres,” Garoua, Cameroon, established in 2007

• Polyclinique Universitaire “Le Bon Samaritain,” A.T.C.P., N’Djamena Sud, Chad, established in 2008

• Centre Medico Social, Eglise Catholique Apostolique Romaine, Ihosy, Madagascar, established in 2008

• Hôpital “Henintsoa,” Vohipeno, Madagascar, established in 2008

• Hospital “Nossa Senhora da Paz,” Compańia de Santa Teresa de Jesus, Cubal, Angola, established in 2011

• A.M.E.N. Centro Médico, Funda, Angola, established in 2012

• Hospital “Saint Damien,” Tabarre, Republic of Haiti, established in 2012

## Galenic medicinal products and administered therapies

The laboratories produce liquid preparations, capsules, ointments, pessaries, and suppositories. The Project also plans to introduce multi-dose parenteral solutions. Medicinal products are made according to specific local needs and based on the WHO list of essential medicines for DCs (14). There is also a specific handbook prepared for each laboratory. More than 100 different formulations can be prepared, as well as pediatric dosages for each product. Pediatric formulations are very important since their availability is limited. The main pharmaceutical forms are suppository and liquid preparations since can be easily modified to adapt to the weight of the child.

Angola and Haiti laboratories required more pediatric formulations because of the large number of young patients. The Saint Damien Hospital on Haiti is the only pediatric hospital on the island and it treats about 80 000 children every year. Considering the high incidence of heart disease on site, a specific handbook was prepared for the Haiti GL, which included a number of preparations for the cardiovascular system.

Preparations for pediatric use produced so far are the following:

• Solutions: captopril, furosemide

• Suspensions: amoxicillin, carbocysteine, chloramphenicol, erythromycin, magnesium and aluminum hydroxide, metronidazole, vitamin B complex

• Syrups: ascorbic acid, carbocysteine, ibuprofen, iron sulfate, paracetamol, potassium canrenoate, propranolol, quinine, ranitidine, salbutamol, vitamin B6

• Drops: nifedipine, quinine, ranitidine, salbutamol, vitamin B6

• Suppositories: paracetamol

The costs of raw materials and equipment since the opening of the first laboratory in 2006 until December 2013 amounted to about 190 000 €. We calculated the exact number of treatments administered considering the medicinal product posology and the exact duration of each treatment (Table 1). For example, in the GL in Chad, with 83 kg of acetylsalicylic acid, the average number of therapies administered each year was 568 lower doses and 15 563 higher doses (the lower dose is 100 mg/cps and the higher dose – 500 mg/cps – is three times more prescribed). In all our laboratories 10 000 000 oral doses were administered.

**Table 1:** The number of capsules prepared in the *A.P.P.A.*^®^ laboratories in the world from 2006 to 2013

Active principle	The number of capsules produced
Acetylsalicylic acid	496,000
Acyclovir	500
Allopurinol	10,000
Aluminum hydroxide + magnesium hydroxide	172,500
Aminophylline	70,000
Amoxicillin	1,374,273
Atenolol	46,667
Bisacodyl	34,000
Captopril	76,000
Carbocysteine	23,600
Chloramphenicol	83,920
Ciprofloxacin	82,667
Diclofenac	428,000
Erythromycin	75,733
Ethambutol + isoniazid	80,000
Folic acid	331,400
Furosemide	261,538
Hydrochlorothiazide	99,000
Ibuprofen	724,167
Iron sulfate + folic acid	478,750
Loperamide	50,000
Mebendazole	185,143
Metformin	94,074
Metoclopramide	20,000
Metronidazole	583,867
Nifedipine	270,000
Nimesulide	36,500
Paracetamol	1,521,176
Praziquantel	4,167
Prednisolone	226,000
Quinine sulfate	242,983
Ranitidine	115,091
Rifampicin + isoniazid	44,667
Salbutamol	1,080,000
Verapamil	1,250
Vitamin B complex (B1 + B2 + B3 + B5 + B6)	288,889
Vitamin B12	133,333
Vitamin C	211,543
TOTAL	10,057,397

## Actions to ensure quality

In order to ensure the quality of the products, which is a prerequisite for their safety and efficacy, galenics were prepared, labeled, and stored according to standard procedures and established methods. The specific operative procedures and handbook for each laboratory mirror specific local needs and guarantee the products' quality (15,16). Operative procedures and handbooks are written in English, French, Portuguese, or other languages to ensure a better understanding and reproducibility of preparations.

The use of written operative procedures and quality controls are required by the European law for the preparation of medicines. The DCs do not generally have a specific legislation related to galenics and if they do, it is extremely deficient and cannot ensure the quality of the products. However, in each new *A.P.P.A.*^®^ laboratory, the head of the health care facility has a duty to inform the local competent authority. Sometimes, the local authorities have specific structural requests. For example, the Government of Madagascar required that each room in the laboratory is dedicated to producing a different type of preparation, eg, solid or liquid.

The control and quality assurance is performed on site and in Italy (the tests applied are listed in Table 2). Each assay is carried out using instruments and methods in compliance with the Official Pharmacopoeia of the European Union (Ph. Eur. 7 ed. (Table 3) (17), although the European standard imposes simpler controls for galenics than those listed and the tests are normally required only for industrial medicines.

**Table 2:** Tests performed for quality assurance and control of galenic medicinal products (16,17)

Tests performed in the laboratories located in DCs	Tests performed in the University of Turin laboratories
Raw materials	
• Organoleptic control • Melting point (Ph. Eur. Monograph 2.2.14) (17)	• Organoleptic control • Melting point (Ph. Eur. Monograph 2.2.14) • Spectrophotometric analysis
**Pharmaceuticals forms**	
**Infusions, liquid preparations for cutaneous application, liquid preparations for oral use, pessaries, hard capsules, semi-solid preparations for cutaneous application, suppositories**	
• Verification of the procedures accuracy • Control of aspect • Control of the product amount aimed for the market • Control of the solidity of packaging	• Uniformity of content (Ph. Eur. Assay 2.9.6)
**Infusions**	
	• Sterility test (Ph. Eur. Assay 2.6.1) • Bacterial endotoxins (Ph. Eur. Assay 2.6.14)
**Liquid preparations for cutaneous application**	
• pH (Ph. Eur. Monograph 2.2.3)	• pH (Ph. Eur. Monograph 2.2.3)
**Liquid preparations for oral use**	
• pH (Ph. Eur. Monograph 2.2.3)	• pH (Ph. Eur. Monograph 2.2.3)
**Pessaries**	
• Uniformity of mass (Ph. Eur. Assay 2.9.5)	• Uniformity of mass (Ph. Eur. Assay 2.9.5) • Disintegration (Ph. Eur. Assay 2.9.2)
**Hard capsules**	
• Uniformity of mass (Ph. Eur. Assay 2.9.5)	• Uniformity of mass (Ph. Eur. Assay 2.9.5) • Disintegration (Ph. Eur. Assay 2.9.1)
**Suppositories**	
• Uniformity of mass (Ph. Eur. Assay 2.9.5)	• Uniformity of mass (Ph. Eur. Assay 2.9.5) • Disintegration (Ph. Eur. Assay 2.9.2)

**Table 3:** Acceptance criteria for quality assurance and control tests of galenic medicinal products (16,17)

Analysis	Method of reference reported in Ph. Eur.	Acceptance criteria
pH	Monograph 2.2.3	The pH does not differ from the expected value
Melting point	Monograph 2.2.14	The melting point does not differ from the value in the monograph
Uniformity of content	Assay 2.9.6	The content is between 85% and 115% of the average content
Uniformity of mass	Assay 2.9.5	Not more than two of the individual masses deviate from the average mass by more than ±10% and none deviates by more than ±20% (20 dosage units)
Disintegration	Assay 2.9.1 Assay 2.9.2	At the end of the specified period all of the dosage units must have disintegrated completely
Sterility test	Assay 2.6.1	At intervals during the incubation period and at its conclusion, examine the media for macroscopic evidence of microbial growth: If no evidence of microbial growth is found, the product complies with the test for sterility
Bacterial endotoxins	Assay 2.6.14	The test is considered valid when all replicates of tested solutions show no reaction and the result of control solution confirms the labeled lysate sensitivity

**Figure 1:**
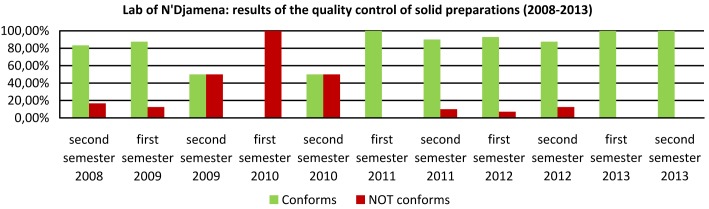
Laboratory in N'Djamena, Chad: results of the quality control of solid preparations (2008-2013).

The results of quality controls are not always positive, as shown on the example from the N’Djamena laboratory (Figure 1). Negative results can be caused by technical and environmental factors. One of the technical factors is insufficient mixing time for powders, which does not allow uniform distribution of the active substance in the capsules. Environmental problems are usually related to atmospheric humidity. Humidity is normally controlled by air conditioners but in DCs the electricity is not always continuously available. Whenever the results of quality controls are not satisfactory, preparatory work stops until the arrival of a member of *A.P.P.A.*^®^ association. After the cause is identified and removed, the checks are repeated and if the outcome is positive, the activity is resumed.

## Stability assessment of galenics

*A.P.P.A.*^®^ performed a survey on the stability of various galenic dosage forms under different environmental conditions in accordance with the EMA guidelines (12).

We gathered information on the stability of galenics in extreme environmental conditions (high temperatures and relative humidity) resembling those in target countries – “accelerated” (*t* = 40 ± 2°C, RH = 60 ± 5%) and “stress” (*t* = 40 ± 2°C; RH = 80 ± 5%) conditions. The main goal was to allow the preservation of preparations in facilities not equipped with air conditioners or refrigerators.

## Selection and training of local staff

The local staff is selected by the head of the health facility. Preferably, they should have working experience in the health field but experience with galenics preparation is not necessary because they undergo training by the *A.P.P.A.*^®^ staff. In addition, in the target countries it is almost impossible to find staff with this kind of background knowledge. The training program is constantly assessed through weekly quizzes and quality control of the prepared medicines. When the local staff proves to be able to apply all the procedures, galenic medicines are administered to the patients.

## The role of Pharmacy students

A distinctive feature of this Project is the involvement of students from the University of Turin, who use the Project as the basis for their thesis preparation. The students undergo an intensive training on technological procedures and the principles of the international health cooperation and their work is constantly monitored by the University of Turin.

## Use of galenics in DCs: economic and social evaluations

The costs of equipment and raw materials are covered by funds raised through the collaboration of groups involved in international cooperation in health. The hospital can also use the funds earned by dispensing the medicinal products to buy raw materials. As a result there will be an uninterrupted production and the laboratory will be self-financed. The sale of galenic products is managed entirely by the health care facility where the laboratory is located.

Galenic sale price on local market is usually equal to the production cost. Raw materials are purchased from the same suppliers for all eight laboratories because they have provided the lowest price without a decrease in raw materials quality. This is why the production cost is always the same regardless of the country.

Galenics are sometimes more expensive than the corresponding industrial medicines available on the local market as those prices are adapted to the purchasing power of the local population. The same Galenic product may be cheaper than the industrial product in one country but not in another. For example, considering the 2013 list price, the cost of an amoxicillin 500 mg capsule produced in an *A.P.P.A.*^®^ laboratory was € 0.04373. The cost of an equivalent industrial product in Chad was € 0.04231 and in Angola € 0.05020. Considering only the production price, the amoxicillin 500 mg capsules were cost-effective in Angola but not in Chad. Generally, the galenic medicines were 100% cost-effective in Angola, 62% in Chad, 73% in Cameroon, and 75% on Madagascar. On Haiti, this evaluation was not applicable because pediatric preparations equivalent to those prepared in the laboratory were not available on-site.

A higher price of the galenic medicinal product compared to the corresponding industrial product could discourage patients to purchase it. In this respect, the health care facility will have the task to sensitize the purchasers about the importance of the quality of medicinal products. In addition *A.P.P.A.*^®^ is responsible to continuously search for quality suppliers that can decrease the costs. It is difficult to achieve the economic autonomy of a GL because in order to obtain a significant gain from the sale of galenics, the hospital should increase the selling prices.

Therefore, different scenarios regarding the economic independence of the GLs can be considered. For example in Cameroon the incomes gained by the sale of galenics are enough to sustain the administration of the “la Bethanie” medical center in Douala. On the contrary, GL in the “Henintsoa” Hospital in Vohipeno on Madagascar still needs donor contributions. Our commitment is also to raise funds through the collaboration of groups involved in the international health cooperation to ensure the continued activities regardless of the economic autonomy.

The current results are satisfactory, but achieving them required overcoming many difficulties. The main difficulties were not scientific but related to bureaucratic and technical problems, such as the electricity or running water supply. Another problem was migration of the local staff, who moved to the richer areas attracted by better economic prospects, which required re-recruiting and training new personnel.

## Conclusions

The Project achieved many goals, the most important of which was to enable local people the access to quality medicinal products in spite of the widespread distribution of counterfeit medicines in DCs. Great satisfaction has also come from the fact that hospital patients and their MDs believed that galenics were more effective than products from street vendors or pharmacies.

According to the principles of international cooperation, the final goal of our Project is that all laboratories become autonomous. Taking into account the results of quality controls and the stable employment of local staff, a laboratory can become technologically independent after about 5 years’ work. Technological independence means that local operators are able to effectively comply with all operative procedures, self-organize the production, and ensure a constant availability of products to meet the hospital's needs.

The effective compliance with the operating procedures was verified through the quality controls by the University of Turin and it can be concluded that respecting the operating procedures largely contributes to satisfactory results. Although the aim of the Project was to promote self reliance and independence, quality controls had to be carried out in Italy due to the cost of the necessary equipment. The external monitoring is important because it ensures a completely objective evaluation of the results.
